# Fabrication, Characterization, and Antioxidant Potential of Sodium Alginate/Acacia Gum Hydrogel-Based Films Loaded with Cinnamon Essential Oil

**DOI:** 10.3390/gels9040337

**Published:** 2023-04-15

**Authors:** Saurabh Bhatia, Ahmed Al-Harrasi, Yasir Abbas Shah, Halima Waleed Khalifa Altoubi, Sabna Kotta, Priyanka Sharma, Md. Khalid Anwer, Deepa Sreekanth Kaithavalappil, Esra Koca, Levent Yurdaer Aydemir

**Affiliations:** 1 Natural and Medical Sciences Research Center, University of Nizwa, Birkat Al Mauz, P.O. Box 33, Nizwa 616, Oman; yasir.shah@unizwa.edu.om (Y.A.S.); 7halima5757@gmail.com (H.W.K.A.); 2 School of Health Science, University of Petroleum and Energy Studies, Dehradun 248007, India; 3 Center for Transdisciplinary Research, Department of Pharmacology, Saveetha Dental College and Hospital, Saveetha Institute of Medical and Technical Sciences, Saveetha University, Chennai 600077, India; 4 Department of Pharmaceutics, Faculty of Pharmacy, King Abdulaziz University, Jeddah 21589, Saudi Arabia; skotta@kau.edu.sa; 5 Center of Excellence for Drug Research and Pharmaceutical Industries, King Abdulaziz University, Jeddah 21589, Saudi Arabia; 6 Center for Innovation in Personalized Medicine, King Fahad Medical Research Center, King Abdulaziz University, Jeddah 21589, Saudi Arabia; aamrahs@kau.edu.sa; 7 Department of Pharmaceutics, College of Pharmacy, Prince Sattam Bin Abdulaziz University, Al-Kharj 11942, Saudi Arabia; m.anwer@psau.edu.sa; 8 Department of Pharmaceutical Sciences, College of Pharmacy, Shaqra University, Shaqra 11961, Saudi Arabia; deepatv@su.edu.sa; 9 Department of Food Engineering, Faculty of Engineering, Adana Alparslan Turkes Science and Technology University, Adana 01250, Turkey; esrakoca.tr@outlook.com (E.K.); lyaydemir@atu.edu.tr (L.Y.A.)

**Keywords:** sodium alginate, hydrogel polymer, acacia gum, cinnamon essential oil, edible hydrogel-based films, food packaging

## Abstract

Several studies have reported the advantages of incorporating essential oils in hydrogel-based films for improving their physiochemical and antioxidant attributes. Cinnamon essential oil (CEO) has great potential in industrial and medicinal applications as an antimicrobial and antioxidant agent. The present study aimed to develop sodium alginate (SA) and acacia gum (AG) hydrogel-based films loaded with CEO. Scanning Electron Microscopy (SEM), X-ray diffraction (XRD), Fourier-transform infrared spectroscopy (FTIR), Differential scanning calorimetry (DSC), and texture analysis (TA) were performed to analyze the structural, crystalline, chemical, thermal, and mechanical behaviour of the edible films that were loaded with CEO. Moreover, the transparency, thickness, barrier, thermal, and color parameters of the prepared hydrogel-based films loaded with CEO were also assessed. The study revealed that as the concentration of oil in the films was raised, the thickness and elongation at break (EAB) increased, while transparency, tensile strength (TS), water vapor permeability (WVP), and moisture content (MC) decreased. As the concentration of CEO increased, the hydrogel-based films demonstrated a significant improvement in their antioxidant properties. Incorporating CEO into the SA–AG composite edible films presents a promising strategy for producing hydrogel-based films with the potential to serve as food packaging materials.

## 1. Introduction

Recent developments in the food packaging industry have revealed that biopolymer-based edible films have great potential to replace plastics. The different characteristics of edible films, such as their non-toxicity, biodegradability, and safety, make them a feasible option for food packaging material [1]. Hydrogels are used in a broad range of applications due to their characteristic properties such as hydrophilicity, non-toxicity, and biocompatibility [2,3,4]. Sodium alginate (SA) is a natural hydrophilic polysaccharide and has been used in the fabrication of biopolymer films due to its excellent film-forming properties [5,6,7]. Sodium alginate-based edible films have demonstrated superior mechanical properties along with good transparency [8]. Sodium alginate is a common hydrogel polymer that tends to form hydrogel via substituting sodium ions of the guluronic acid residues. Despite their good mechanical properties, edible films made from sodium alginate are limited by their high hydrophilicity and poor heat stability [9]. Combining SA with various other polymers is one approach that has been investigated extensively to overcome these challenges.

Acacia gum, also known as gum Arabic, is a gummy exudate obtained from the branches and trunk of the Acacia senegal and Acacia seyal plant species [10]. AG is a water-soluble, highly branched polysaccharide extensively used as a thickener, stabilizer, and emulsifier in the food industry [11]. It has been employed in the production of edible films due to its biocompatibility, renewability, non-toxicity, pH stability, low cost, high solubility, and gelling properties [12]. AG can be used to make edible films; however, it poses several challenges, such as its poor mechanical attributes, its high hydrophilicity, and its poor barrier features [13]. However, the preparation of composite films consisting of SA and AG could be an option to enhance the physiochemical properties of the resulting films. Over the past few years, numerous studies have highlighted the advantages of incorporating bioactive compounds such as essential oils in edible films for improved physiochemical and antioxidant attributes [14,15,16]. In the current study, cinnamon essential oil (CEO) was incorporated in the SA–AG composite hydrogel-based films due to its vast industrial and medicinal applications, such as antimicrobial and antioxidant agents [17]. The essential oil of cinnamon has a variety of significant chemical constituents, the most prominent of which are the compounds aldehyde and alcohol, along with trans-cinnamaldehyde and eugenol [17,18,19]. Zhou et al. [20] studied the physiochemical properties of cassava starch-based edible films that were loaded with cinnamon essential oil. After the assessment, it was found that the films’ barrier properties, crystallinity, and elongation at break (EAB) were considerably enhanced and that there was also an improvement in their thermal stability [20].

Given its antimicrobial and antioxidant characteristics, cinnamon is widely acknowledged as a safe food preservative [21]. The objective of the current study is to assess the physiochemical and antioxidant properties of composite hydrogel-based films based on SA and AG, which have been loaded with varying concentrations of cinnamon essential oil.

## 2. Results and Discussion

### 2.1. Visual Assessment of the Films

**Figure 1:**
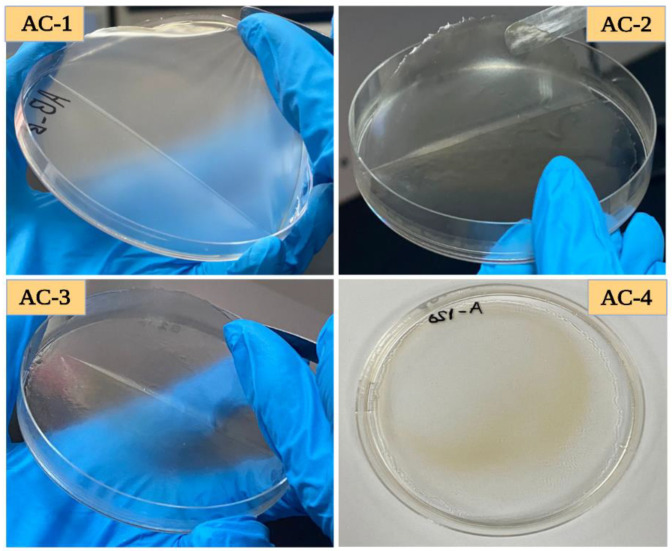
Visual assessment of SA–AG-based composite edible films; AC-1/Control, AC-2 contains 15 μL of CEO, AC-3 contains 20 μL of CEO, and AC-4 contains 30 μL of CEO.

The visual assessment of edible films is necessary to determine the overall quality, appearance, and uniformity of the film structure. This is a crucial aspect for visually assessing hydrogel-based films in terms of their functionality and acceptance by consumers. The visual appearance of the films can impact the consumer perception of the product’s quality and visual defects such as tears, pores, poor mechanical strength, and inconsistencies in thickness. The visual appearance of the SA–AG composite edible films loaded with CEO is shown in Figure 1. In general, as the amount of CEO in the film increased, a less transparent appearance of the film was observed in comparison with the control film. The incorporation of oil resulted in a slightly yellow appearance of the films. The control film sample showed good uniformity and was more transparent compared with film samples that were loaded with oil. However, the CEO-loaded films had good flexibility and physical attributes compared with the control. Moreover, different factors can impact the visual characteristics of the films such as the film composition, the preparation technique, handling, and storage conditions.

### 2.2. Thickness of the Films

The thickness of edible films can vary depending on the type of material and the process used for the preparation. The composite hydrogel-based films based on SA and AG were examined for thickness measurement and the results are presented in Table 1. The control (AC-1) showed minimum thickness (0.065mm) compared with the samples loaded with CEO. The incorporation of the CEO in films significantly increased the thickness from 0.065 to 0.11 mm, with the maximum thickness in the AC-4 sample containing the maximum concentration (30 μL) of the CEO, followed by AC-3 and AC-2. The increase in the thickness could be due to the increase in the viscosity of the film-forming solution with the addition of CEO. Zhou et al. [20] also reported similar results, where the thickness of cassava starch-based films increased when the concentration of the CEO increased. Thickness also affects other parameters, such as mechanical and barrier properties, as well as the transparency and visual appearance of the films. Therefore, the thickness of biopolymer-based edible films is an important factor that needs to be carefully controlled during the preparation of the films to achieve optimal properties for their intended application.

### 2.3. Mechanical Properties of the SA–AG-Based Films

Hydrogel-based films can be prepared from various materials such as proteins, carbohydrates, and lipids that have different mechanical properties. The mechanical properties of the edible film can be modified by changing the composition of the film or the processing conditions. In the current study, TS and EAB parameters were assessed to evaluate the mechanical properties of the SA–AG composite edible films. The TS of the edible films showed a significant decrease (9.82–3.49 Mpa) when increasing the concentration of the CEO. The minimum TS (3.49 Mpa) was observed in the AC-4 film sample with a maximum concentration (30 μL) of CEO, while the blank AC-1 had the maximum value (9.82 Mpa) (Table 1). Wu et al. [22] showed similar results, where the TS of the gelatin-based films decreased when increasing the concentration of the CEO. The tensile strength can be influenced by several factors such as polymer/plasticizer/other additive type and proportion, as well as their respective interaction with each other.

The results of the EAB of the analyzed SA–AG-based film samples are shown in Table 1. The EAB of the SA–AG-based films significantly increased from 7.57 to 18.41% with the addition of the CEO. The maximum and the minimum EAB values were found in AC-4 and AC-1, respectively. The increase in the EAB of the hydrogel-based films could be due to the addition of the CEO, as the oil acts as a plasticizer and makes films more flexible and less brittle. The results of the present study are in line with Wu et al. [23], who reported an increase in the EAB with the addition of CEO nanoliposomes in the gelatin films. Moreover, different factors affect the EAB of the edible films such as the concentration of the added bioactive compounds, the composition of the film-forming solution, and the conditions and method used for the preparations of films.

### 2.4. Moisture Content

The moisture content in edible films is an important factor to consider as it can affect the quality, safety, and shelf life of the food. Generally, the moisture content should be low to prevent microbial growth and maintain the structural integrity of the film. In the current study, the SA–AG-based film samples showed a slight decrease from 18.52% to 17.03% in moisture content with the addition of the CEO. The minimum MC (17.03%) was found in the AC-4 sample, followed by AC-3 (17.89%) and AC-2 (18.18%), while the maximum value for MC was observed in the control. The decrease in the moisture content of the films when increasing the concentration of the CEO could be due to the hydrophobic nature of the added oil. Jamróz et al. [24] reported a decrease in the moisture content of edible films, based on starch-turbellarian-gelatin, when incorporated with tea tree essential oil. Furthermore, sodium alginate-chitosan-based edible films showed similar behaviour when loaded with bitter orange oil [25].

### 2.5. Water Vapor Permeability

In edible films, water vapor permeability (WVP) significantly impacts the shelf life and the quality of the packed food product. The WVP of an edible film is dependent on the properties of the film-forming material, such as its composition and structure. The environmental conditions, such as the temperature and humidity of the storage environment, can also affect the WVP of the hydrogel-based films. A lower WVP is desirable for certain applications as it slows down moisture migration and increases the shelf life of the product. The water vapor permeability of the SA–AG composite films significantly decreased with the addition of the CEO (Table 1). The maximum WVP (0.424) was exhibited by the control compared with the film samples loaded with the CEO. The AC-4 sample showed the minimum WVP (0.353), followed by the AC-3 (0.390) and AC-2 (0.405) samples. The decrease in the WVP could be due to the hydrophobicity of the CEO, which resulted in better barrier properties for the hydrogel-based films regarding water vapor permeability. The results of the current study are in line with the findings of Suput et al. [26] and Sánchez-González et al. [27]. Furthermore, the WVP of the edible films also depends on the type of polymers used, the concentration of the oil, and the preparation technique and conditions.

### 2.6. Transparency and Color Parameters

Transparency and color parameters are important factors as they affect the visual appearance, freshness, and quality parameters of food products. In the current study, the SA–AG composite hydrogel films incorporated with the CEO were examined for transparency and color parameters, including Lightness (L), a*, b*, and delta E. The addition of oil had a significant impact on the transparency of the films (Table 2). The transparency decreased from 79 to 21% with the addition of oil; the maximum was observed in the control (AC-1) and the minimum was shown by the AC-4 sample, which contained the maximum concentration of the CEO. The results of the current study are in accord with the findings of a previous study, in which the transparency of starch-based films decreased with the addition of oregano and black cumin essential oil [26].

The SA–AG based films showed a slight decrease in Lightness (L), from 96.05 to 91.64%, with the addition of oil. The b* value of the films varied from 0.94 to 4.97, whereases the a* value ranged from 0.08 to 0.26. As mentioned in Table 2, the films showed (b*) yellowness as the concentration of the CEO increased, with a maximum b* value (4.97) in the AC-4 sample. The significant variation of ΔE values (0.85–5.04) also validates the overall color alterations in the films with the addition of oil. Essential oils contain a complex mixture of polyphenolic components that tends to absorb light, which can ultimately impact the color attributes of the film. Zhou et al. [20] reported a similar behavior in which the color of the cassava starch-based films changes to yellow with the addition of cinnamon essential oil. Furthermore, Tongnuanchan et al. [28] and Atarés et al. [29] reported similar results.

### 2.7. Scanning Electron Microscopy

**Figure 2:**
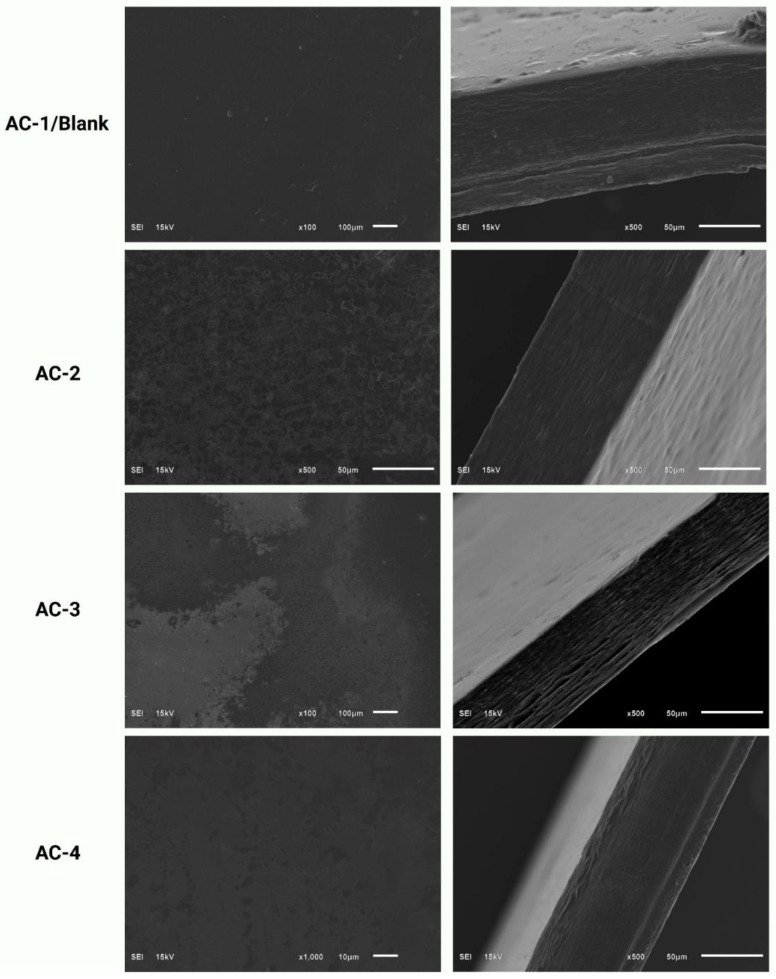
Scanning Electron Microscopy of SA–AG hydrogel-based edible films; AC-1/Control, AC-2 contains 15 μL of CEO, AC-3 contains 20 μL of CEO, and AC-4 contains 30 μL of CEO.

Scanning Electron Microscopy (SEM) is a technique that is used to observe the surface structure of materials at high magnification. In the case of edible films, SEM is used to study their physical characteristics, such as their microstructure, uniformity, compactness and surface texture. SEM imaging can also help to identify any defects in the film, such as cracks, pores, or voids, that could impact its functionality and performance as food packaging. The SEM results of the SA–AG film samples with the added CEO are shown in Figure 2. The control film (AC-1) containing SA and AG without the CEO showed a structure with some pores on the film surface, compared with the films loaded with CEO. The protrusion can also be observed in the cross-section image of the AC-1 sample. The randomly distributed oil drops can be observed on the film surface of the AC-2 sample. The roughness of the AC-2 film samples could be linked to the agglomeration that resulted from the irregular distribution of hydrophobic components during the film-forming procedure. The most uniform dispersion was observed in the AC-4 sample, which contained the maximum concentration of the CEO. The film samples incorporated with the CEO showed smooth surfaces with a fewer number of particles, homogeneous structures, and no pores and cracks. Zhou et al. [20] also reported similar results, in which the CEO distributed homogeneously in the polymer matrix of cassava starch-based films. Moreover, the interaction between oil and the film-forming polymers can also influence the SEM appearance, as some polymers may be more compatible with oil than others.

### 2.8. X-ray Diffraction Analysis

**Figure 3:**
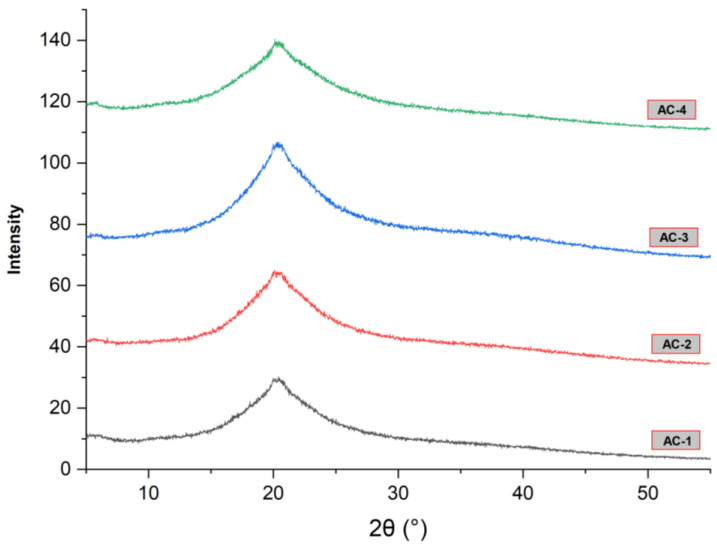
X-ray diffraction pattern of SA–AG hydrogel-based edible films; AC-1/blank, AC-2 contained 15 μL of CEO, AC-3 contained 20 μL of CEO, and AC-4 contained 30 μL of CEO.

XRD analysis of edible films is important because it provides important information about the structure and composition of the material. XRD provides information about the crystalline structure of edible films, which provides insight into their mechanical properties, such as their tensile strength and flexibility. The SA–AG hydrogel-based composite films loaded with the CEO were examined for their structural characteristics and the resulting spectrum is shown in Figure 3. A Diffract Eva software package was used to calculate the crystallinity percentages of the film samples, and the crystallinity percentages of the AC-1, AC-2, AC-3, and AC-4 samples were found to be 38.1%, 38.7%, 35.4%, and 38.6%, respectively. All the samples showed characteristic peaks at similar positions with different intensities due to the variations in the concentration of the oil. However, there was no difference observed in the crystallinity of the SA–AG composite hydrogel films with the addition of the CEO. Overall, the XRD patterns showed good compatibility between the film-forming polymers including SA, AG and CEO.

### 2.9. Fourier Transform Infrared Spectroscopy

**Figure 4:**
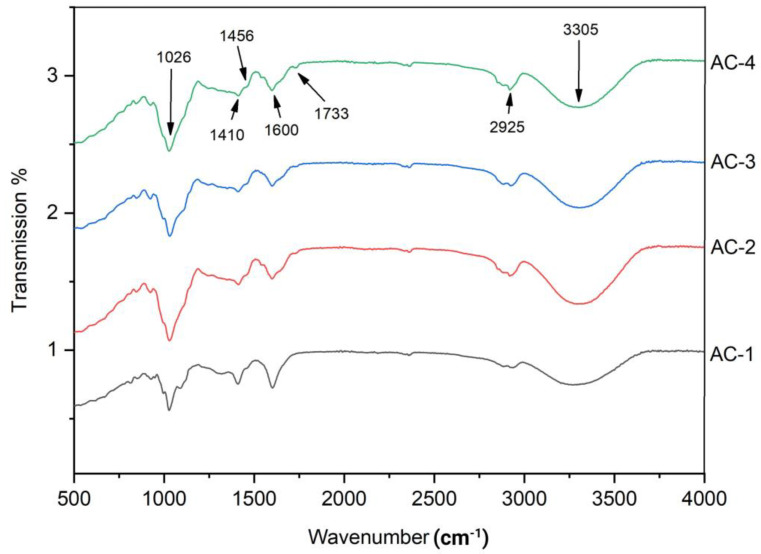
FTIR spectrum of SA–AG hydrogel-based edible films; AC-1/Control, AC-2 contains 15 μL of CEO, AC-3 contains 20 μL of CEO and AC-4 contains 30 μL of CEO.

FTIR provides information about the molecular structure and composition of the film, it also provides valuable information about the degree of cross-linking in the film, which affects its mechanical properties and durability. SA and AG composite edible films loaded with the CEO were analyzed for FTIR and the absorption spectrum is shown in Figure 4. The broad spectrum at 3305 cm^−1^ indicates the stretching vibration of the secondary N-H amide bonds [30]. The characteristic peaks identified at 1410 cm^−1^ and 1600 cm^−1^ could have been due to the presence of sodium alginate in the film matrix, representing the symmetric and asymmetric stretching vibration of the COO-group respectively [31]. Additionally, studies have also reported that characteristic peaks identified at 1400 cm^−1^ and 2925 cm^−1^ positions could be due to the presence of sodium alginate [25]. In a previous study, it was reported that AG exhibited a characteristic peak at 2929 cm^−1^ in the FTIR analysis [32]. A study reported the -C=O stretching vibration of the tween 80 carbonyl group at 1735 cm^−1^ and 1736 cm^−1^ during the FTIR analysis [20]. In our current study, the film matrix exhibited a characteristic peak at 1733 cm^−1^ that could be ascribed to the presence of tween 80 in the film matrix; however, a little variation (from 1735 cm^−1^ to 1733 cm^−1^) could have been due to the difference in the concentration. A distinctive peak at 1456 cm^−1^ corresponding to the phenolic group of cinnamaldehyde was observed in the FTIR spectrum that indicates the presence of CEO in the film matrix [20]. Overall, the FTIR analysis showed a good intermolecular interaction between the CEO, SA and AG.

### 2.10. DSC Analysis

**Figure 5:**
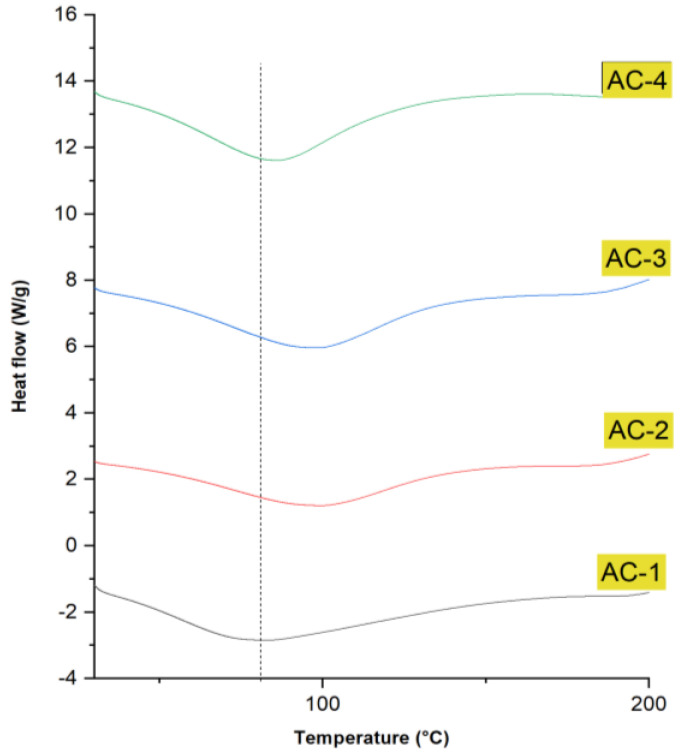
DSC analysis of SA–AG-based composite edible films; AC-1/Control, AC-2 contains 15 μL of CEO, AC-3 contains 20 μL of CEO, and AC-4 contains 30 μL of CEO.

The thermal stability of edible films refers to their ability to maintain their physical, chemical, and structural properties when exposed to elevated temperatures. Thermal stability is a critical factor for the performance and functionality of edible films, as it affects their shelf life and suitability for various food packaging applications. DSC analysis can be used to evaluate the thermal stability of edible films by measuring the temperature and heat flow during heating or cooling cycles. The DSC thermograms of the SA–AG-based composite films are shown in Figure 5. The AC-2, AC-3, and AC-4 films incorporated with CEO presented one broad endothermic peak at 70–128 °C, 72–126 °C, and 51–118 °C, respectively. This endothermic peak can be attributed to the evaporation of the residual solvent (water) that was used during the production of composite films [33,34]. After the incorporation of oil into the films, the temperature of the endothermic peak increased significantly in AC-2 and AC-3 samples, but the temperature slightly reduced in the AC-4 sample with a maximum (30 μL) of CEO. As the concentration of CEO was increased, the peak area of the AC-2, AC-3, and AC-4 samples also increased, indicating that the thermal stability of the composite films improved due to the addition of EOs. This shift could have been due to the plasticization effect of oil, which would have raised the free volume within the polymeric network and mobility of the polymeric chains as reported in the previous studies [35].

The improved thermal stability of the composite hydrogel-based films indicates that there were strong intermolecular interactions between the CEO and composite material, which could potentially influence the mechanical properties of the films. The previous studies suggested that the incorporation of *Origanum vulgare* L. and *Matricaria recutita* essential oil caused a change in endothermic peaks, indicating the thermal stability of the polymeric films [36,37].

As per previous reports, the incorporation of *Origanum onites* L. essential oil reduced the heat transitions due to evaporation, as evidenced by changes in endothermic peaks. This could be due to the molecular structure of the essential oil that caused the changes in the overall chain mobility of the polymer matrix, as reported in previous studies [38,39].

### 2.11. Antioxidant Potential

**Figure 6:**
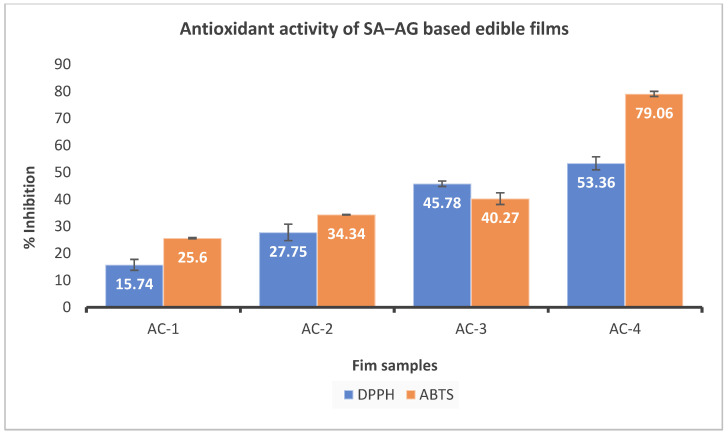
Antioxidant activity of SA–AG-based composite edible films; AC-1/Control, AC-2 contains 15 μL of CEO, AC-3 contains 20 μL of CEO, and AC-4 contains 30 μL of CEO.

The antioxidant activity of hydrogel-based films can help to protect the food packaged within the film from oxidation, which can cause the food to spoil or lose quality. The addition of essential oils to edible films can increase their antioxidant potential, and can help to protect the packaged food from oxidation and extend its shelf life. Essential oils are concentrated plant extracts that contain a variety of antioxidants, such as phenolic compounds, terpenes, and flavonoids [40]. In the current study, the antioxidant activity of the different samples of edible films based on SA and AG was assessed and the results are shown in Figure 6. The hydrogel-based films showed a significant increase in the DPPH radical scavenging activity with increasing the concentration of the CEO. Similar results were obtained for the ABTS radical scavenging activity of the SA–AG-based composite film samples. The films incorporated with CEO exhibited more antioxidant activity compared with the control. This could have been due to the presence of different bioactive compounds in the CEO, primarily cinnamaldehyde, contributing to the overall antioxidant potential of the edible films [41]. Xu et al. [42] found similar results in which the antioxidant activity of the chitosan-gum arabic films increased with the addition of CEO. Furthermore, many studies have reported an increase in the antioxidant activity of edible films when incorporated with essential oils [43,44,45].

## 3. Conclusions

The current study revealed that CEO has the potential to be used for the development of sodium alginate and acacia gum-based composite films with improved physicochemical properties at optimal concentrations. The findings of the study are likely to be a valuable resource for the development of future edible packaging formulations, as well as for identifying potential applications for different food products. However, further investigations can reveal the impact of adding the CEO on the antimicrobial properties of sodium alginate-acacia gum-based films.

## 4. Materials and Methods

### 4.1. Film-Formation

SA and AG were provided by Sisco Research Laboratories (SRL), Mumbai, India. 1.5% (*w*/*v*) solution of SA and a 0.5% (*w*/*v*) solution of AG were prepared separately by dissolving the polymers in distilled water and stirring them overnight for complete solubilization at the magnetic stirrer. After complete solubilization, both solutions were subjected to mixing with the gradual addition of 1% glycerol (BDH Laboratory, London, England) as a plasticizer. The resultant solution was subjected to stirring for 3 h at a magnetic stirrer. The obtained film-forming solution was divided equally into four beakers and labelled as AC-1, AC-2, AC-3, and AC-4. Subsequently, different concentrations (15 μL, 20 μL and 30 μL) of the CEO (Nature Natural, Ghaziabad, India) were added to AC-2, AC-3, and AC-4 samples, respectively, while the AC-1 sample without the addition of the CEO was used as a control sample. Additionally, different concentrations of 30 μL, 40 μL, and 80 μL of tween 80 (Sisco Research Laboratories, Mumbi, India) were also added as a surfactant to AC-2, AC-3, and AC-4 samples, respectively, for the uniform dispersion of oil in the film-forming solution. The obtained solutions were poured onto the labelled petri plates and left to dry for 48 h at room temperature. After drying, the films were evaluated visually and subjected to further examination.

### 4.2. Thickness

To determine the thickness of the films produced, a digital micrometre (Yu-Su 150, Yu-Su Tools, China) was utilized. The thickness measurements were taken at 5 distinct positions of the film and an average was calculated.

### 4.3. Mechanical Properties

The mechanical properties of the hydrogel films, specifically their tensile strength (TS) and percentage elongation at break (EAB), were determined using a texture analyzer (XT plus, Stable Micro Systems, Godalming, England) following the standard ASTM D882 method [46]. For the present test, film strips that were 60 mm long and 7 mm wide were used. The equations presented below were used to determine the values of the TS and EAB for the film samples.

(1) $$\mathrm{TS}=\left(\frac{F}{A}\right)$$

F is the maximum force,

A is the cross-sectional area of the film.

(2) $$\mathrm{EAB}\;\left(\%\right)=\frac{\mathrm{Lf}-\mathrm{Li}}{\mathrm{Li}}\times 100$$

Lf is the final length at a break, 

Li is the initial length of the film.

### 4.4. Moisture Content

The hydrogel film strips (3 cm × 4 cm) were examined for their moisture content (MC). The film strips were dried at 105 °C and the difference in weight was calculated before (W1) and after drying (W2), according to Equation (3).

(3) $$\mathrm{MC}=\frac{W1-W2}{W1}\times 100$$

### 4.5. Water Vapor Permeability

The procedure followed by Erdem et al. [47] was used to measure the water vapor permeability (WVP) of the films. The relative humidity (RH) of the apparatus was adjusted to 100% and 0% by using water and silica gel, respectively. Hydrogel films were sealed firmly over the glass test cups (5cm of internal diameter and 3 cm depth) containing silica gel. The cups were weighed at specific intervals to calculate the weight-gain within a day. Equation (4) was used to calculate the WVP of the films and represented in g mm/(m^2^)(d)(kPa).

(4) $$\mathrm{WVP}=\frac{\Delta m}{\Delta t\times \Delta P\times A}\times d$$

In Equation (4), the ∆m/∆t is the weight of moisture-gain per unit of time. A is the film area in m^2^; ∆P is the water-vapor pressure difference between the two sides of the film in kPa; d is the film thickness in mm.

### 4.6. Transparency and Color Parameters

The transparency of the SA–AG hydrogel film samples was measured at a wavelength of 550 nm by using a spectrophotometer (ONDA-Vis spectrophotometer, V-10 Plus, ONDA, Padova, Italy) according to the method described by Zhao et al. [48].

The surface color analysis of the SA–AG-based films was carried out by using a colorimeter (Konica Minolta, Tokyo, Japan), and represented as L* (lightness), a* (red/green), and b* (yellow/blue). The film samples were placed on the surface of a standard plate (L* = 100) and the color parameters L*, a*, and b* were calculated. Equation (5) was used to calculate the ΔE (the overall color difference).

(5) $${\Delta E}^{*}={\left[{\left({\Delta L}^{*}\right)}^{2}+\left({\Delta a}^{*}\right){\;}^{2}+{\left({\Delta b}^{*}\right)}^{2}\right]}^{1/2}$$

### 4.7. Microstructure of the Films

The microstructure analysis of the prepared hydrogel film samples based on SA–AG was carried out by using Scanning Electron Microscopy (SEM) (JSM6510LA from Analytical SEM, Jeol, Tokyo, Japan). The films were initially mounted on double-sided tape on an aluminum stub that was coated with a thin layer of gold before the analysis.

### 4.8. X-ray Diffraction (XRD) Analysis

To determine the crystallinity of the samples, X-ray diffractometer (Bruker D8 Discover) was used by applying 40 kV voltage with 2 theta ranging from 5–50° at a rate of 0.500 s/point, employing copper (Kα) radiation (1.5418 Å).

### 4.9. Fourier Transforms Infrared Spectroscopy (FT-IR)

FTIR analysis of the SA–AG hydrogel-based films was performed by using an InfraRed Bruker Tensor 37, Ettlingen, Germany. The obtained spectrum was used to examine the functional groups and the interactions between film-forming components. The test was performed with an average of 32 scans, with a wavenumber range from 400 to 4000 cm^−1^.

### 4.10. Differential Scanning Calorimetry

DSC measurements were performed using DSC-Q20 instrument (TA instruments, New Castle, DE, USA). 10 mg of film was hermetically encapsulated in aluminum capsules and placed in the sampling unit. The sample was heated from 25 °C to 200 °C at a heating rate of 10 °C/min in a nitrogen-rich atmosphere.

### 4.11. Antioxidant Analysis

The antioxidant potential of the SA–AG-based films was analyzed using DPPH and ABTS radical scavenging activities. For the DPPH assay, the method described by Brand-Williams et al. [49] was followed to carry out the analysis for 50 mg of film samples. The absorbance was recorded at 517 nm using an ONDA-Vis spectrophotometer. The obtained results were presented as % inhibition. For the ABTS assay, the methodology of Re et al. [50] was employed to evaluate the antioxidant activity of the 25 mg of film samples. The absorbance was recorded at 734 nm and the results that were obtained were presented as % inhibition as an average of three measurements. The films were directly treated with radical solutions, vortexed for 30 s and incubated for radical scavenging activity.

### 4.12. Statistical Analysis

All data in the current study are reported as the mean and standard deviation (SD) of three distinct assessments. A one-way analysis of variance was conducted using statistical analysis software, followed by Duncan’s test with a 5% significant level. The purpose of these analyses was to examine whether the mean values differed significantly.

## Figures and Tables

**Table 1:** The thickness, tensile strength, elongation at break, moisture content and water vapor permeability of SA–AG hydrogel-based films.

Film Sample	Thickness (mm)	Tensile Strength (Mpa)	Elongation at Break (%)	Moisture Content (%)	Water Vapor Permeability ((g * mm)/(m^2^ * h * kPa))
AC-1	0.065 ± 0.006 ^a^	9.82 ± 0.41 ^a^	7.57 ± 0.45 ^a^	18.52 ± 0.41 ^a^	0.424 ± 0.018 ^a^
AC-2	0.075 ± 0.006 ^a^	7.31 ± 0.65 ^b^	12.24 ± 0.60 ^b^	18.18 ± 0.39 ^ab^	0.405 ± 0.007 ^a^
AC-3	0.098 ± 0.005 ^b^	5.59 ± 0.27 ^c^	14.94 ± 0.79 ^c^	17.89 ± 0.59 ^bc^	0.390 ± 0.015 ^b^
AC-4	0.110 ± 0.006 ^c^	3.49 ± 0.02 ^d^	18.41 ± 0.60 ^d^	17.03 ± 0.08 ^c^	0.353 ± 0.004 ^c^

Means carrying the same letters are significantly identical (*p* > 0.05).

**Table 2:** Transparency and color parameters of SA–AG hydrogel-based films.

Film Sample	Transparency (%)	L	a*	b*	ΔE*
AC-1	79.99 ± 0.62 ^a^	96.05 ± 0.12 ^a^	−0.08 ± 0.02 ^a^	0.94 ± 0.08 ^a^	0.85 ± 0.09 ^a^
AC-2	73.85 ± 2.04 ^b^	94.40 ± 0.07 ^b^	0.03 ± 0.03 ^b^	2.68 ± 0.11 ^b^	2.98 ± 0.10 ^b^
AC-3	64.02 ± 1.05 ^c^	92.39 ± 0.30 ^c^	0.05 ± 0.02 ^b^	3.42 ± 0.47 ^c^	4.36 ± 0.43 ^c^
AC-4	21.23 ± 2.27 ^d^	91.64 ± 0.15 ^d^	0.26 ± 0.05 ^c^	4.97 ± 0.52 ^d^	5.04 ± 0.54 ^c^

Means carrying the same letters are significantly identical (*p* > 0.05). L: lightness, a*: green-red color, b*: blue-yellow color, ΔE*: overall color variation.

## Data Availability

Not applicable.

## References

[1] Dhumal C.V., Sarkar P. (2018). Composite edible films and coatings from food-grade biopolymers. J. Food Sci. Technol..

[2] Ibrahim A.G., Abdel Hai F., Abd El-Wahab H., Aboelanin H. (2018). Methylene blue removal using a novel hydrogel containing 3-Allyloxy-2-hydroxy-1-propanesulfonic acid sodium salt. Adv. Polym. Technol..

[3] Ibrahim A.G., Sayed A.Z., Abd El-Wahab H., Sayah M.M. (2020). Synthesis of a hydrogel by grafting of acrylamide-co-sodium methacrylate onto chitosan for effective adsorption of Fuchsin basic dye. Int. J. Biol. Macromol..

[4] Elkony A.M., Ibrahim A.G., Abu El-Farh M.H., Abdelhai F. (2021). Synthesis of Acrylamide-co-3-Allyloxy-2-hydroxy-1-propanesulfonic acid sodium salt Hydrogel for efficient Adsorption of Methylene blue dye. Int. J. Environ. Anal. Chem..

[5] Galus S., Lenart A. (2013). Development and characterization of composite edible films based on sodium alginate and pectin. J. Food Eng..

[6] Gheorghita R., Gutt G., Amariei S. (2020). The use of edible films based on sodium alginate in meat product packaging: An eco-friendly alternative to conventional plastic materials. Coatings.

[7] Mahcene Z., Khelil A., Hasni S., Akman P.K., Bozkurt F., Birech K., Goudjil M.B., Tornuk F. (2020). Development and characterization of sodium alginate based active edible films incorporated with essential oils of some medicinal plants. Int. J. Biol. Macromol..

[8] Dou L., Li B., Zhang K., Chu X., Hou H. (2018). Physical properties and antioxidant activity of gelatin-sodium alginate edible films with tea polyphenols. Int. J. Biol. Macromol..

[9] Liu S., Li Y., Li L. (2017). Enhanced stability and mechanical strength of sodium alginate composite films. Carbohydr. Polym..

[10] Sanchez C., Nigen M., Tamayo V.M., Doco T., Williams P., Amine C., Renard D. (2018). Acacia gum: History of the future. Food Hydrocoll..

[11] Suresh S.N., Puspharaj C., Natarajan A., Subramani R. (2022). Gum acacia/pectin/pullulan-based edible film for food packaging application to improve the shelf-life of ivy gourd. Int. J. Food Sci. Technol..

[12] Ibrahim A.G., Elkony A.M., El-Bahy S.M. (2021). Methylene blue uptake by gum arabic/acrylic amide/3-allyloxy-2-hydroxy-1-propanesulfonic acid sodium salt semi-IPN hydrogel. Int. J. Biol. Macromol..

[13] Kang S., Xiao Y., Guo X., Huang A., Xu H. (2021). Development of gum arabic-based nanocomposite films reinforced with cellulose nanocrystals for strawberry preservation. Food Chem..

[14] Pelissari F.M., Grossmann M.V., Yamashita F., Pineda E.A.G. (2009). Antimicrobial, mechanical, and barrier properties of cassava starch− chitosan films incorporated with oregano essential oil. J. Agric. Food Chem..

[15] Gómez-Estaca J., De Lacey A.L., López-Caballero M., Gómez-Guillén M., Montero P. (2010). Biodegradable gelatin–chitosan films incorporated with essential oils as antimicrobial agents for fish preservation. Food Microbiol..

[16] Wu Y., Luo Y., Wang Q. (2012). Antioxidant and antimicrobial properties of essential oils encapsulated in zein nanoparticles prepared by liquid–liquid dispersion method. LWT-Food Sci. Technol..

[17] Nwanade C.F., Wang M., Wang T., Zhang X., Zhai Y., Zhang S., Yu Z., Liu J. (2021). The acaricidal activity of cinnamon essential oil: Current knowledge and future perspectives. Int. J. Acarol..

[18] Wang R., Wang R., Yang B. (2009). Extraction of essential oils from five cinnamon leaves and identification of their volatile compound compositions. Innov. Food Sci. Emerg. Technol..

[19] Deng X., Liao Q., Xu X., Yao M., Zhou Y., Lin M., Zhang P., Xie Z. (2014). Analysis of essential oils from cassia bark and cassia twig samples by GC-MS combined with multivariate data analysis. Food Anal. Methods.

[20] Zhou Y., Wu X., Chen J., He J. (2021). Effects of cinnamon essential oil on the physical, mechanical, structural and thermal properties of cassava starch-based edible films. Int. J. Biol. Macromol..

[21] Gooderham N.J., Cohen S.M., Eisenbrand G., Fukushima S., Guengerich F.P., Hecht S.S., Rietjens I.M., Rosol T.J., Davidsen J.M., Harman C.L. (2020). FEMA GRAS assessment of natural flavor complexes: Clove, cinnamon leaf and West Indian bay leaf-derived flavoring ingredients. Food Chem. Toxicol..

[22] Wu J., Sun X., Guo X., Ge S., Zhang Q. (2017). Physicochemical properties, antimicrobial activity and oil release of fish gelatin films incorporated with cinnamon essential oil. Aquac. Fish..

[23] Wu J., Liu H., Ge S., Wang S., Qin Z., Chen L., Zheng Q., Liu Q., Zhang Q. (2015). The preparation, characterization, antimicrobial stability and in vitro release evaluation of fish gelatin films incorporated with cinnamon essential oil nanoliposomes. Food Hydrocoll..

[24] Jamróz E., Juszczak L., Kucharek M. (2018). Development of starch-furcellaran-gelatin films containing tea tree essential oil. J. Appl. Polym. Sci..

[25] Bhatia S., Al-Harrasi A., Al-Azri M.S., Ullah S., Bekhit A.E.-D.A., Pratap-Singh A., Chatli M.K., Anwer M.K., Aldawsari M.F. (2022). Preparation and physiochemical characterization of bitter Orange oil loaded sodium alginate and casein based edible films. Polymers.

[26] Suput D., Lazic V., Pezo L., Markov S., Vastag Z., Popovic L., Rudulovic A., Ostojic S., Zlatanovic S., Popovic S. (2016). Characterization of starch edible films with different essential oils addition. Pol. J. Food Nutr. Sci..

[27] Sánchez-González L., Vargas M., González-Martínez C., Chiralt A., Cháfer M. (2009). Characterization of edible films based on hydroxypropylmethylcellulose and tea tree essential oil. Food Hydrocoll..

[28] Tongnuanchan P., Benjakul S., Prodpran T. (2013). Physico-chemical properties, morphology and antioxidant activity of film from fish skin gelatin incorporated with root essential oils. J. Food Eng..

[29] Atarés L., Bonilla J., Chiralt A. (2010). Characterization of sodium caseinate-based edible films incorporated with cinnamon or ginger essential oils. J. Food Eng..

[30] Marzbani P., Resalati H., Ghasemian A., Shakeri A. (2016). Surface modification of talc particles with phthalimide: Study of composite structure and consequences on physical, mechanical, and optical properties of deinked pulp. BioResources.

[31] Chan H., Nyam K., Yusof Y., Pui L. (2020). Investigation of properties of polysaccharide-based edible film incorporated with functional melastoma malabathricum extract. Carpathian J. Food Sci. Technol..

[32] Venkatesham M., Ayodhya D., Madhusudhan A., Veerabhadram G. (2012). Synthesis of stable silver nanoparticles using gum acacia as reducing and stabilizing agent and study of its microbial properties: A novel green approach. Int. J. Green Nanotechnol..

[33] Altiok D., Altiok E., Tihminlioglu F. (2010). Physical, antibacterial and antioxidant properties of chitosan films incorporated with thyme oil for potential wound healing applications. J. Mater. Sci. Mater. Med..

[34] Kaya M., Khadem S., Cakmak Y.S., Mujtaba M., Ilk S., Akyuz L., Salaberria A.M., Labidi J., Abdulqadir A.H., Deligöz E. (2018). Antioxidative and antimicrobial edible chitosan films blended with stem, leaf and seed extracts of Pistacia terebinthus for active food packaging. RSC Adv..

[35] Sobral P.d.A., Menegalli F., Hubinger M., Roques M. (2001). Mechanical, water vapor barrier and thermal properties of gelatin based edible films. Food Hydrocoll..

[36] Hosseini S.F., Rezaei M., Zandi M., Farahmandghavi F. (2015). Bio-based composite edible films containing Origanum vulgare L. essential oil. Ind. Crops Prod..

[37] Aliheidari N., Fazaeli M., Ahmadi R., Ghasemlou M., Emam-Djomeh Z. (2013). Comparative evaluation on fatty acid and Matricaria recutita essential oil incorporated into casein-based film. Int. J. Biol. Macromol..

[38] Scartazzini L., Tosati J., Cortez D., Rossi M., Flôres S., Hubinger M., Di Luccio M., Monteiro A. (2019). Gelatin edible coatings with mint essential oil (*Mentha arvensis*): Film characterization and antifungal properties. J. Food Sci. Technol..

[39] Qin Y., Li W., Liu D., Yuan M., Li L. (2017). Development of active packaging film made from poly (lactic acid) incorporated essential oil. Prog. Org. Coat..

[40] Magalhaes M., Manadas B., Efferth T., Cabral C. (2021). Chemoprevention and therapeutic role of essential oils and phenolic compounds: Modeling tumor microenvironment in glioblastoma. Pharmacol. Res..

[41] Suryanti V., Wibowo F., Khotijah S., Andalucki N. Antioxidant activities of cinnamaldehyde derivatives. Proceedings of the IOP Conference Series: Materials Science and Engineering.

[42] Xu T., Gao C., Feng X., Yang Y., Shen X., Tang X. (2019). Structure, physical and antioxidant properties of chitosan-gum arabic edible films incorporated with cinnamon essential oil. Int. J. Biol. Macromol..

[43] Tongnuanchan P., Benjakul S., Prodpran T. (2014). Comparative studies on properties and antioxidative activity of fish skin gelatin films incorporated with essential oils from various sources. Int. Aquat. Res..

[44] Tongnuanchan P., Benjakul S., Prodpran T. (2012). Properties and antioxidant activity of fish skin gelatin film incorporated with citrus essential oils. Food Chem..

[45] Pires C., Ramos C., Teixeira B., Batista I., Nunes M., Marques A. (2013). Hake proteins edible films incorporated with essential oils: Physical, mechanical, antioxidant and antibacterial properties. Food Hydrocoll..

[46] Properties A.S.D.o.M. Standard Test Method for Tensile Properties of Thin Plastic Sheeting. https://www.wewontech.com/testing-standards/190125019.pdf.

[47] Erdem B.G., Dıblan S., Kaya S. (2019). Development and structural assessment of whey protein isolate/sunflower seed oil biocomposite film. Food Bioprod. Process..

[48] Zhao J., Wang Y., Liu C. (2022). Film Transparency and Opacity Measurements. Food Anal. Methods.

[49] Brand-Williams W., Cuvelier M., Berset C. (1995). Antioxidative activity of phenolic composition of commercial extracts of sage and rosemary. LWT.

[50] Re R., Pellegrini N., Proteggente A., Pannala A., Yang M., Rice-Evans C. (1999). Antioxidant activity applying an improved ABTS radical cation decolorization assay. Free Radic. Biol. Med..

